# The Cross-Talk Between the Heart and the Liver: The Involvement of the Mitral Valve as a Novel Actor upon the Ancient Scene of Liver Cirrhosis

**DOI:** 10.3390/jcdd12020076

**Published:** 2025-02-17

**Authors:** Domenico Cozzolino, Riccardo Nevola, Alberto Ruggiero, Ciro Romano, Giuseppina Rosaria Umano, Ernesto Aitella, Celestino Sardu, Aldo Marrone, Sandro Gentile

**Affiliations:** 1 Department of Precision Medicine, “Luigi Vanvitelli” University of Campania, 80131 Naples, Italy; domenico.cozzolino@unicampania.it; 2 Liver Unit, AORN S. G. Moscati, “A. Landolfi” Hospital, 83029 Solofra, Italy; riccardo.nevola@aornmoscati.it; 3 Cardiology Unit, S. Anna and S. Sebastiano Hospital, 81100 Caserta, Italy; albertoruggiero@aorncaserta.it; 4 Department of Advanced Medical and Surgical Sciences, “Luigi Vanvitelli” University of Campania, 80131 Naples, Italy; celestino.sardu@unicampania.it (C.S.); aldo.marrone@unicampania.it (A.M.); 5 Department of the Woman, Child, and General and Specialized Surgery, “Luigi Vanvitelli” University of Campania, 80138 Naples, Italy; giuseppinarosaria.umano@unicampania.it; 6 Department of Clinical Medicine, Public Health, Life and Environmental Sciences, University of L’Aquila, 67100 L’Aquila, Italy; ernestoaitella@gmail.com; 7 Nefrocenter Research, 80141 Naples, Italy; s.gentile1949@gmail.com

**Keywords:** liver cirrhosis, Child–Pugh score, echocardiography, mitral valve structure and function

## Abstract

Background: To date, little is known about correlations between liver dysfunction and circulatory and cardiac abnormalities (e.g.,: mitral valve, MV) in patients with chronic liver disease (CLD). This study aimed to assess a potential parallelism between liver dysfunction and cardiovascular involvement and identify the factors associated with structural and functional MV disorders. Methods. Among 995 patients with CLD, 346 were enrolled and compared with 168 controls without liver disease. According to the degree of liver disease, patients were classified as patients with chronic hepatitis (142) or with liver cirrhosis (Child-A: 70; Child-B: 65; Child-C: 69). Results: Among the chronic hepatitis group, resting heart rate (HR) and left ventricular (LV) mass were higher than in the control group (*p* = 0.0008), whereas systemic vascular resistance (SVR) was lower (*p* = 0.01). Among cirrhotic patients, resting HR, left atrium dimensions/volumes, LV walls thickness, LV mass, cardiac output (CO), isovolumetric relaxation time (IVRT), deceleration time (DT) and prevalence of aortic stenosis were higher than in non-cirrhotic patients (*p* = 0.02), whereas the e/a ratio and SVR were lower (*p* = 0.0001). Among Child-B/C, CO, IVRT, DT, prevalence of MV regurgitation and MV calcification score were higher than in the remaining patients (*p* = 0.02), whereas SVR was lower (*p* < 0.0001). Among cirrhotic patients with MV regurgitation, Child–Pugh score, liver disease duration, resting HR, left chambers dimensions/mass, CO, IVRT, DT and MV calcification score were higher compared to patients without regurgitation (*p* < 0.000), whereas mean blood pressure, e/a ratio and SVR were lower (*p* = 0.008). At multivariate analysis, Child–Pugh score, liver disease duration, left chambers volume/mass and MV calcification score were independently associated with MV regurgitation in cirrhotic patients. Child–Pugh score and MV calcification score strongly correlated in cirrhotic patients (r = 0.68, 95% CI 0.60–0.75, *p* < 0.0001). Conclusions: The magnitude of cardiac morpho/functional abnormalities is associated with the severity of liver dysfunction. Structural and functional MV abnormalities could represent a novel sign of cardiac involvement in liver cirrhosis. The severity and duration of liver disease, the enlargement of cardiac chambers and leaflet calcium accumulation could play a key role.

## 1. Introduction

Since the original work by Kowalski and Abelman that first described cardiac involvement in patients affected by liver cirrhosis, a number of circulatory and cardiac abnormalities have been reported in these patients [1]. Circulatory changes observed in these patients recall a condition of hyperdynamic circulatory state. This condition is essentially characterised by high cardiac output (CO), a decrease in systemic vascular resistance (SVR), an increase in heart rate (HR) at rest and an expansion of total blood volume secondary to sodium and water retention [2]. In this context, a key role is played by an abnormal vasodilatation, which is sustained, on the one hand, by an increase in several circulating substances, including nitric oxide, prostaglandins and endo-cannabinoids and, on the other hand, by the hyporeactivity of smooth muscular and endothelial cells to vasoconstrictor factors, including angiotensin-II and endothelin-1 [3]. Furthermore, a latent autonomic nervous dysregulation responsible for an altered blood pressure regulation during dynamic conditions is operative in liver cirrhosis [4]. A few years ago, the term cirrhotic cardiomyopathy (CC) was coined to indicate a constellation of several structural and functional cardiac aberrancies, mainly represented by the structural derangement of cardiac chambers, an impaired contractile response of the left ventricle (LV) to physiological and pharmacological stressors, LV diastolic dysfunction and electrophysiological disorders [5,6]. One of the first autoptic studies conducted in about 200 hospitalised patients dying with cirrhosis documented a significant increase in LV mass and a substantial enlargement of cardiac chambers [7]. More recently, interest in identifying eventual additional heart abnormalities in patients with liver cirrhosis is increasing, and much attention has been focused on coronary vessels and cardiac valves in cirrhotic patients. Coronary catheterisation-based studies in patients with liver dysfunction have demonstrated a significantly greater prevalence of coronary artery disease than in the general population [8]. Similarly, a 2–5% chance of severe aortic stenosis and a ~1% chance of severe aortic regurgitation have been estimated in patients with liver cirrhosis [9]. One of the hypothetical mechanisms leading to such a severe derangement of coronary vessels and cardiac valves in liver cirrhosis could be represented by aberrancy in calcium homeostasis and a tendency to abnormal calcium tissue deposition. In fact, abnormalities in calcium metabolism have been reported in patients with advanced liver disease, irrespective of the coexistence of hepatocarcinoma/cholangiocarcinoma, hyperparathyroidism and/or hypovitaminosis D [10].

To date, while the data regarding functional and/or structural changes of cardiac chambers, aortic valve and coronary vessels in patients with liver cirrhosis are numerous irrespective of the degree of liver dysfunction, little is known about the involvement of the mitral valve (MV) in this disease, and equally little is known about the possible parallelism between the severity of any cardiac abnormality and the degree of hepatopathy in these patients. Furthermore, it is not completely clear whether there is or is not any involvement of the heart in patients suffering from chronic hepatitis, a long-lasting condition which often precedes the development of cirrhosis later in life, irrespective of the aetiology responsible for liver disease. The present study was conducted in a large cohort of patients with chronic liver disease consecutively admitted to our Unit and rigorously classified on the basis of own liver disease stage and aimed: (i) to evaluate the systemic haemodynamics and structure and function of left cardiac chambers and valves, with a particular focus on MV; (ii) to speculate on a supposed tendency of cardiac abnormalities to worsen in parallel with the progression of liver disease; and (iii) to identify the factors/conditions potentially implicated in the development of structural and functional MV disorders in the setting of cirrhotic patients.

## 2. Materials and Methods

### 2.1. Subjects

Between 1 January 2006 and 31 December 2019, 995 patients with chronic liver disease were consecutively admitted to our Department of Internal Medicine at University of Campania L. Vanvitelli, Naples. Italy. Of these, 346 patients were enrolled in the study, whereas the remaining 649 were excluded because of one or more of the following exclusion criteria: severe anaemia (Hb < 7 g/dL), end-stage liver failure (MELD score > 10), severe hepatic encephalopathy, ultrasound evidence of severe ascites, liver cancer, diabetes mellitus, kidney failure, hydro-electrolyte abnormalities, history of systemic hypertension, coronary artery disease (on the basis of history, signs and symptoms consistent with ischemic heart disease), hyperlipidaemia, other major cardiovascular disorders (including major rhythm disorders), lung, haematological or systemic diseases and use of inotropic and antiarrhythmic drugs during the previous 30 days. Based on clinical, laboratory, instrumental and/or histological features, chronic hepatitis was diagnosed in 142 patients (CH group; 30 M/112 F), and liver cirrhosis in the remaining 204 patients (124 M/80 F). According to the Child–Pugh scoring system, cirrhotic patients were classified as Child A (n 70; 46 M/24 F), Child B (n 65; 43 M/22 F) and Child C (n 69; 35 M/34 F). The aetiology of liver disease among CH and cirrhotic patients is reported in Table 1. A group of 168 (84 M/84 F) subjects hospitalised for minor diseases served as controls. Fifteen days before testing, diuretics, beta-blockers and calcium channel blockers were discontinued in all patients. All patients were on an isocaloric and low-sodium (daily sodium intake: 20–25 mmol) diet. All subjects enrolled in the study gave their written informed consent to participate in this observational study, after a clear and detailed explanation of its nature. All patients were subjected only to routine care practice; no experimental procedures were carried out. The study was conducted according to the guidelines of the Declaration of Helsinki and its later amendments. Finally, the study was carried out at the teaching hospital of the “Luigi Vanvitelli” University of Campania as a spin-off of a previous protocol (no. 12771/i).

### 2.2. Methods

Patients’ history, physical examination and routine laboratory and instrumental investigations were collected on in-hospital admission day, whereas haemodynamic and echocardiographic examinations were performed at established times. Routine laboratory assays were performed inside our institution by available commercial kits.

After 7 days (at least) of in-hospital bed rest, each subject was studied after an overnight fast, between 08.00 h and 10.00 h A.M. All subjects were initially equilibrated for 45 min in a thermoneutral (26–27 °C) room while lying supine before haemodynamic and echocardiographic evaluations were obtained. As already described, each subject was positioned in a supine position while HR, blood pressure and SVR were continuously and non-invasively monitored by a beat-to-beat monitor (CC-NEXFIN, BMEYE NA, Amsterdam, The Netherland). Data acquisition started at least 30 min after instrumentation and continued for a period of 15 min [11]. Mean values of the parameters recorded were used for calculations.

All patients underwent transthoracic echocardiography according to the standards recommended [12] by using an ultrasound mechanical system equipped with a 2.5–3.5 MHz transducer (HD11 XE Philips Medical Systems, Amsterdam, The Netherland). An electrocardiographic tracing was displayed simultaneously as the ultrasound images. LV diastolic function was evaluated by measuring the e/a ratio, isovolumetric relaxation time (IVRT) and deceleration time (DT) of the e wave. LV mass was determined according to Devereux’s formula and was indexed to height to the power of 2.7 [13]. LV ejection fraction (LVEF) was computed from apical two- and four-chamber views, using the modified Simpson biplane method. Aiming to obtain a complete/exhaustive anatomical and functional MV assessment, several echo scanning planes were considered, and the parasternal (long- and short-axis) and apical (four-, two-, and three-chamber) windows were used [14]. In addition, much care was taken to evaluate and quantify any eventual calcium deposition on the MV leaflets. For these purposes, the grading of MV leaflet calcification was assessed according to the semiquantitative/qualitative Wilkins scoring system [15], which allowed us to categorise each subject as follows: grade 1 = evidence of a single area of increased leaflet echo brightness (one point); grade 2 = evidence of scattered areas of leaflet echo brightness, confined to leaflet margins (two points); grade 3 = evidence of echo brightness of margins extending into the mid-portion of the leaflets (three points); grade 4 = evidence of extensive brightness throughout much of the leaflet tissue (four points). Much care was taken to have minimal angulation between the ultrasound beam and the plane of cardiac motion. All echocardiographic measurements were calculated from an average of five consecutive cardiac cycles. In our laboratory, the intra-observer mean coefficient of variations of the main parameters considered, as assessed in 25 healthy subjects by repeating the measurements 14 days apart, were <5%. The inter-observer mean coefficients of variation of the same parameters were <5%. All images were stored and analysed by two experienced cardiologists blinded to the subjects’ clinical details.

### 2.3. Statistical Analysis

All the analyses have been performed using SAS^®^ University Edition (SAS Institute Inc., Cary, NC, USA). Differences for continuous variables among the groups were assessed by one-way ANOVA. Post-hoc analysis for multiple comparisons was performed by applying Bonferroni correction. Chi-square tests were performed to evaluate differences in categorical variables, and Yates’ correction was applied when appropriate. Among the three groups of cirrhotic patients, some variables which demonstrated association (*p* < 0.05) with MV regurgitation after a comparison of characteristics of the patients with and without MV regurgitation were included in the univariate analyses. Univariate logistic regression analyses were performed to identify candidate variables for multivariate analysis to estimate the risk of MV regurgitation. Statistically significant variables at the univariate analysis have been included in the multivariate analysis. The Spearman rank correlation (r) procedure was used to examine the individual relationship between MV calcification score according to the Wilkins scoring system and Child–Pugh score among cirrhotic patients. Data were expressed as mean ± SD. All statistical tests were two-sided. Significance was considered with *p* < 0.05.

## 3. Results

The male-to-female ratio was heterogeneous, whereas the mean age of the groups was substantially superimposable, except for Child B patients who were significantly more aged (*p* = 0.003, Table 1). Weight in patients affected by liver disease was significantly lower than in control counterparts (*p* < 0.0001), and it was significantly lower in the Child C group than in the other groups of patients with liver disease (*p* = 0.02, Table 1). Body mass index in cirrhotic patients was significantly lower than in non-cirrhotic patients (*p* = 0.007, at least) and was significantly lower in both Child C and B patients compared to Child A cirrhotic patients (*p* < 0.0001, Table 1). Liver disease duration in cirrhotic patients was significantly longer than in CH patients (*p* = 0.0006, at least) and significantly longer in Child C patients than in the remaining cirrhotic patients (*p* < 0.0001, Table 1). Values of circulating albumin in cirrhotic patients were significantly lower than in control subjects and CH patients (*p* = 0.005, at least); they were significantly lower in Child B patients than in Child A patients (*p* = 0.01) and significantly lower in Child C patients compared to the remaining cirrhotic patients (*p* = 0.04 at least, Table 1). Serum creatinine in patients with liver disease (except for Child A patients) was significantly greater than in control subjects (*p* = 0.005, at least) and significantly higher in Child B patients than in CH patients (*p* = 0.01) and in Child C patients than in the others (*p* < 0.0001, Table 1). Plasma bilirubin values in cirrhotic patients were significantly higher than in controls (*p* = 0.0004, at least), in Child B patients than in CH patients (*p* = 0.01) and in Child C patients compared to both CH and Child A patients (*p* = 0.04 at least, Table 1). B-type natriuretic peptide circulating levels in both Child B and Child C patients were significantly higher than in the remaining patients (*p* < 0.001 at least, Table 1). Mean blood pressure in the Child C group was significantly lower than in the other groups of patients (*p* < 0.0001, Table 1).

Resting HR in patients with liver disease was significantly higher than in control subjects (*p* = 0.0008, at least). It was significantly higher in both the Child A and Child B groups compared to CH patients (*p* = 0.006, at least), and in Child C patients than in the remaining patients with liver disease (*p* < 0.0001, Table 1). When both indexed to body surface area and not, the left atrium in cirrhotic patients was significantly larger than in both control subjects and CH patients (*p* < 0.0001) and in Child C patients when compared to the other cirrhotic counterparts (*p* = 0.01 at least, Table 2). No differences in LV posterior wall thickness were found among the groups except for Child B patients, whose values were significantly greater when compared to both controls and CH patients (*p* = 0.003, at least, Table 2). Interventricular septum thickness in cirrhotic patients was significantly greater than in both controls and CH patients (*p* = 0.003 at least, Table 2). LV diastolic volume indexed to body surface area in Child B and Child C patients was significantly larger than in control subjects (*p* = 0.03, at least) and in Child C than in CH patients (*p* = 0.03, Table 2). Both LV systolic volume and ejection fraction were not significantly different by comparing the groups of subjects/patients of our cohort (Table 2). Indexed LV mass in patients with liver disease was significantly higher than in controls (*p* < 0.0001), in cirrhotic patients than in CH patients (*p* = 0.006, at least) and in the Child C group when compared to the remaining cirrhotic patients (*p* < 0.0001, Table 2).

CO in cirrhotic patients was significantly greater than in non-cirrhotic patients (*p* = 0.009, at least), and in both Child B and Child C patients than in Child A counterparts (*p* < 0.0001, Table 2). The e/a ratio in cirrhotic patients was significantly lower than in non-cirrhotic patients (*p* = 0.004, at least) and in Child C patients than in Child A ones (*p* < 0.0001, Table 2). IVRT in cirrhotic patients was significantly longer than in non-cirrhotic subjects (*p* < 0.0001), in Child B than in Child A patients (*p* < 0.0001) and in Child C patients when compared to the remaining cirrhotic patients (*p* < 0.0001, Table 2). In analogy, DT in cirrhotic patients was significantly longer than in non-cirrhotics (*p* < 0.0001), in Child B patients when compared to Child A patients (*p* < 0.0001) and in Child C patients than in the remaining cirrhotic patients (*p* < 0.0001, Table 2). The prevalence of aortic valve regurgitation was not significantly different between the five groups, whereas the prevalence of aortic valve stenosis in cirrhotic patients was significantly higher than in non-cirrhotic patients (*p* = 0.02 at least, Table 2). The prevalence of MV regurgitation in the Child B and Child C groups of patients was significantly higher than in the remaining groups (*p* = 0.02 at least, Table 2). Grading of mitral valve regurgitation severity in our cirrhotic patients ranged between mild (56 patients; ~50.4%) and moderate (54 patients; ~48.6%), except for one patient from the Child C group who exhibited a severe grade of valve insufficiency. By sub-analysing the Child A, Child B and Child C patient groups, 20 (~66%), 20 (~50%) and 16 (~38%) patients, respectively, showed a mild grade of mitral valve regurgitation, whereas moderate valve regurgitation was found in 10 (~34%), 19 (~50%) and 25 (~60%) patients, respectively, with significant differences comparing the groups in both cases (*p* = 0.008, at least). There were no significant differences in the prevalence of MV stenosis comparing the groups (Table 2). The prevalence of MV calcification in Child B and Child C cirrhotic patients was significantly greater than in the remaining non-cirrhotic patients (*p* < 0.03 at least, Table 2). In analogy, MV calcification score in Child B and Child C patients was significantly higher than in the remaining patients (*p* = 0.001 at least, Table 2). Indirectly calculated on the basis of tricuspid regurgitation jet velocity, estimated values of pulmonary artery systolic pressure at rest in Child B and Child C patients were significantly (*p* = 0.02, at least) higher than in the remaining patients (Table 2). SVR was significantly lower in patients with liver disease than in controls (*p* = 0.01, at least), in cirrhotic patients than in CH patients (*p* = 0.0001, at least) and in both Child B and Child C patients than in Child A patients (*p* < 0.0001, Table 2).

Based on echocardiographic findings, cirrhotic patients were divided into two subgroups: with (n 111; 54%) and without (n 93; 46%) MV regurgitation (Table 3). Patients with MV regurgitation exhibited significantly greater values of Child–Pugh score, liver disease duration, serum creatinine, total bilirubin, HR, indexed left atrial volume, indexed LV mass, CO, IVRT, DT and MV calcification score compared to patients without MV regurgitation (*p* = 0.008, at least), whereas plasma albumin, mean blood pressure, e/a ratio and SVR were significantly lower (*p* = 0.008 at least, Table 3).

According to the univariate analysis, the conditions associated with MV regurgitation were higher values of Child–Pugh score, liver disease duration, total bilirubin, HR, left atrial volume, LV mass, CO, IVRT and MV calcification score and lower values of plasma albumin, mean blood pressure, e/a ratio and systemic vascular resistance (Table 4). The multivariate analysis showed that factors independently associated with MV regurgitation were higher values of Child–Pugh score, liver disease duration, left atrial volume, LV mass and MV calcification score, whereas an independent association between a prolonged isovolumetric relaxation time and MV regurgitation was only slight (Table 4). Finally, a strong positive correlation between Child–Pugh score and MV calcification score (r = 0.68, 95% CI 0.60–0.75, *p* < 0.0001) was found among cirrhotic patients.

## 4. Discussion

The present study mainly evaluated haemodynamics and functional/structural aspects of left cardiac chambers and valves in a large cohort of patients with chronic liver disease irrespective of aetiology responsible for hepatic disease, with a special focus on MV structure and function. Furthermore, an eventual parallelism between the degree of some heart abnormalities and the stage of liver dysfunction has been speculated on. For these purposes, a group consisting of patients suffering from chronic hepatitis, a condition known to precede by years the development of liver cirrhosis in most cases, and a group of cirrhotic patients, rigorously selected and categorised in three subgroups on the basis of Child–Pugh score, were recruited. Our CH patients showed no significant differences when compared to control subjects, except for an increase in LV mass, a decrease in SVR and, as opposed, an expected increase in resting HR. Some of these findings could represent a systemic cardiovascular effect of chronic hepatitis and could be, at least in part, attributable to an altered pathway of nitric oxide and/or a maladaptation of endothelial cells to this molecule, both as a consequence of a low-grade chronic stimulation on the liver exerted by proinflammatory cytokines [16,17,18]. As expected, cirrhotic patients in the present study exhibited abnormal circulating levels of albumin, creatinine and bilirubin, and their values were linearly altered in accordance with the clinical severity of liver disease as expressed by the Child–Pugh scoring system. There was an analogous trend in haemodynamic parameters in our cohort of cirrhotic patients. Indeed, both arterial pressure and SVR in patients with liver cirrhosis were found to be lower than in the remaining patients, whereas a proportionate baroreceptor-mediated increase in HR detected in these patients has been interpreted as a compensatory force in an attempt to warrant a balanced circulatory homeostasis. From a practical point of view, this condition summarises the so-called cirrhotic hyperdynamic circulatory syndrome, where several vasodilating circulating substances (*in primis* nitric oxide) cause a decrease in effective circulating volume mediated by a substantial reduction in mesenteric and systemic arterial resistance, which in turn activates the sympathetic nervous system and renin–angiotensin–aldosterone system [19].

Noteworthily, haemodynamic differences were significant not only comparing cirrhotic patients to non-cirrhotic individuals but also comparing cirrhotic patients with more severe liver disease, especially Child C patients, to those with a lesser degree of liver dysfunction, namely Child A patients. A significant enlargement of both left cardiac chambers during diastole was found in our cirrhotic patients compared to non-cirrhotic patients, whereas LV systolic volume and ejection fraction were not significantly different. Similarly, estimated LV mass in patients with liver cirrhosis were significantly greater than in other individuals in the present study.

Statistical analysis of these data revealed significant differences by comparing the groups of cirrhotic patients and found that patients with a higher grade of liver compromise, according to Child–Pugh scoring, showed more serious abnormalities among echocardiographic parameters evaluated. In analogy, CO was found to be abnormally increased in our cirrhotic patients, especially in the Child B and Child C groups, when compared to the remaining patients despite a superimposable LV systolic function at rest. Notably, the majority of structural and morphological disorders found in the present study are in line with previous observations. An autopsy-based study of patients with liver cirrhosis showed cardiomegaly, slight hypertrophy, interstitial oedema and fibrosis in most patients [20]. A previous study conducted in eighty patients with post hepatitis C virus (HCV) liver cirrhosis reported a significant increase in LV wall thickness and mass and left atrial and ventricular volumes when compared to matched controls [21]. In addition, a recent study conducted in cirrhotic patients and based on cardiac magnetic resonance imaging, a widely diffuse technique able to provide better sensitivity and a lesser intra-operator variability when compared to ultrasonography, described significantly higher values of atrial volumes, CO, LV relaxation time and interstitial oedema compared to control subjects and a strong relationship between the degree of myocardial oedema and the clinical stage of liver cirrhosis [22]. In the present study, cirrhotic patients showed a condition of abnormal LV diastolic function. Furthermore, abnormalities in these parameters tended to be even more evident in the groups of cirrhotic patients with more severe degrees of liver dysfunction. These latter anomalies together with the previous ones, above described, all fall within the so-called CC, where, by definition, systolic–diastolic dysfunction of the left ventricle, hyperdynamic circulation and electrical abnormalities represent the hallmarks of CC. In the present study, circulating levels of B-type natriuretic peptide, one of the biochemical markers suggestive of clinical/subclinical cardiac dysfunction, were elevated in cirrhotic patients, especially among those with more severe liver dysfunction. There is evidence that such markers are directly related to liver disease decompensation [23]. It has been postulated that at least some of the abovementioned structural and functional aspects of CC could be ascribed to a systemic pro-inflammatory state sustained by abnormally elevated levels of some circulating substances, including tumour necrosis factor (TNF)-α, endocannabinoids and nitric oxide [24,25]. In fact, a previous anatomy-based study by Wiese conducted in patients with end-stage liver cirrhosis described an increase in myocardial fibrosis and an abnormal expansion of extracellular volume in myocardium, both as an expression of myocardial scarring secondary to a long-lasting inflammatory state [26]. In addition, an altered signal transduction and down-regulation of the cardiomyocyte β-adrenoreceptors secondary to increased plasma levels of catecholamines have been hypothesised to be implicated in the complex pathogenesis of mechanical heart dysfunction in liver cirrhosis, especially during stress [27].

In agreement with previous reports, the aortic valve has been found to be involved in patients with liver cirrhosis in the present study. Indeed, the prevalence of aortic valve stenosis in the pool of cirrhotic patients was found to be about double that of non-cirrhotic patients, whereas the prevalence of aortic valve regurgitation was not significantly different comparing the groups. However, aligned with previous works, mild-to-moderate aortic regurgitation and stenosis were evidenced in ~12.5% and ~6%, respectively, of our cirrhotic patients [28,29]. No patient exhibited severe aortic valve regurgitation, whereas one patient in the Child–Pugh B group showed severe aortic valve stenosis.

To our knowledge, the present study is the first to heavily focus on MV involvement in patients affected by liver cirrhosis. Taken together, the findings of the present study suggest an unequivocal MV functional and structural involvement in liver cirrhosis. Firstly, the prevalence of MV regurgitation in our patients with more severe liver dysfunction was higher than in other counterparts, whereas the prevalence of MV stenosis was not statistically different comparing the groups. Regurgitation of the mitral valve in patients with liver cirrhosis depends on a number of factors, all more less equally contributing. Other than mitral valve structural derangement due to abnormal leaflet calcium deposition, valve annular dilatation and leaflets prolapse, both secondary to LV remodelling, left chamber size enlargement and an elongation of the papillary muscles and chordae tendineae, on the one hand, and volume overload and hyperdynamic circulatory state, secondary to neuro-hormonal hyperactivation, on the other hand, could play a critical role. Accordingly, dysfunction of the mitral valve among cirrhotic patients in the present study was more severe in subsets of subjects with more serious liver compromise. In order to evaluate the grading of MV calcification, the Wilkins scoring system, a non-invasive semiquantitative/qualitative method able to quantify even the smallest leaflet calcium deposits and widely used by cardiac surgeons as a pre-operative evaluation of patients affected by MV stenosis when surgery intervention is planned, has been adopted in the present study. Notably, the prevalence of MV leaflet calcification in cirrhotic patients with a more severe grade of liver dysfunction was more than 90% and significantly higher than in the remaining patients recruited in this study. Moreover, the entity of MV calcification in patients with more advanced stage of liver disease was found to be greater than in the other groups of the present study. It is conceivable that a hyperdynamic circulatory state, responsible for the mechanical injury of the endocardium, together with a disturbed calcium metabolism might overexpose cardiac valves to the pathological accumulation of calcium deposits in cirrhotic patients. Hypercalcemia and a possible tendency to metastatic tissue deposition have been described in patients with liver cirrhosis, especially in those with decompensated disease. Several mechanisms, including immobilisation, rise in circulating inflammatory substances and unbalance of the RANKL/osteoprotegerin system, have been suggested to be at least in part responsible for the resorption of bone and subsequent hypercalcemia in these patients [30,31,32]. Moreover, a strong association has recently been demonstrated between metastatic tissue calcifications and decrease in the hepatic production of inorganic pyrophosphate, a physiological anti-calcifying compound, due to liver dysfunction in cirrhotic patients [33]. In addition, due to a decrease in the circulation of 1,25(OH)2 vitamin D secondary to a compromised liver production of vitamin D 25-hydroxylase, calcium intestinal absorption is reduced, and secondary hyperparathyroidism develops, which, in turn, favours cardiovascular metastatic calcifications in patients with liver cirrhosis [34].

Almost all the parameters evaluated in the present study, including laboratory, liver disease degree and duration, left chamber mass and function, haemodynamics and MV calcification score, among cirrhotic patients with MV regurgitation were frankly worse than among those without MV regurgitation. At univariate analysis, MV regurgitation was found to be significantly associated with higher Child–Pugh score, liver disease duration, abnormal levels of plasma albumin and bilirubin, a decrease in blood pressure, an increase in resting HR, higher size/mass of left chambers, higher CO, abnormal LV diastolic function, higher MV calcification score and reduced SVR. At multivariate analysis, higher Child–Pugh score, longer disease duration, larger size of cardiac left chambers and higher MV calcification score were found to be independent factors associated with MV regurgitation among patients with liver cirrhosis, whereas IVRT was only slightly associated with MV regurgitation. As expected, functional and structural abnormalities of MV leaflets are in some way linked to the severity of liver dysfunction, which, in turn, is strictly dependent on disease duration: indeed, a strong and positive correlation was found between Child–Pugh score and MV calcification score. However, there is evidence that for many of the variables examined in the present study, the greater the severity of liver dysfunction, the greater the degree of almost all haemodynamic and cardiac abnormalities among our cirrhotic patients, suggesting a hypothetical tendency towards a parallel evolution of cardiovascular abnormalities and of worsening liver impairment. From a practical point of view, given the increasing prevalence of liver cirrhosis worldwide and assuming that cardiac valves and vessels are often compromised in this disease, any effort should be made by physicians to identify cardiac anomalies, to optimise any eventual treatment and, finally, to establish the best timing of heart valve repair, when necessary, in these patients. Furthermore, given the well-known fragility of these patients, especially among those with end-stage liver dysfunction, it is important to clarify the most appropriate intervention strategy aiming to address heart valve dysfunction/derangement in cirrhotic patients: better to repair or to substitute?

There are some limitations to the present study. Firstly, one bias could be represented by the relatively small sample size of our cohort of patients with liver disease. To better understand any connection between the degree of liver dysfunction and the severity of cardiac changes among cirrhotic patients, a dedicated randomised follow-up work is needed in the future. This study is essentially based on a qualitative non-invasive technique, and the grading of valve calcification has been evaluated by means of a scoring system according to Wilkins. It is likely that some quantitative techniques, such as computed tomography and/or magnetic resonance imaging, known to be more accurate (but more expensive), might have provided a more punctual assessment of heart valvular changes, compared to the less expensive and more available echocardiography utilised in the present study. In addition, at least some of the cirrhotic patients might be suffering from a concomitant condition of portopulmonary hypertension, but the structure and function of the right ventricle, including the pulmonary and tricuspid valves, have not been evaluated in this study.

## 5. Conclusions

In conclusion, the findings of the present study have shown only slight haemodynamic changes and no involvement of the heart among patients with chronic hepatitis, irrespective of aetiology. Morphological and functional changes in cardiac left chambers together with a hyperdynamic circulatory state have been confirmed in cirrhotic patients. The structure and function of the mitral valve have been found to be altered in patients with liver cirrhosis, and leaflet calcification and valvular regurgitation represent the main features. The degree of almost all these abnormalities were found to be related to the severity of liver dysfunction. The severity and duration of liver disease, the enlargement of cardiac left chambers and the degree of leaflet calcium accumulation are all factors strongly associated with mitral valve dysfunction.

## Figures and Tables

**Table 1:** Main clinical characteristics in control subjects, patients with chronic hepatitis (CH) and patients with cirrhosis categorised on the basis of Child–Pugh score.

	Control Group (no. 168)	CH Group (no. 142)	Child A Group (no. 70)	Child B Group (no. 65)	Child C Group (no. 69)	*p*
Sex (M/F)	84/84	30/112	46/24	43/22	35/34	-
Age (yrs)	62.3 ± 12.2	62.2 ± 9.7 *	63.6 ± 10.6	65.7 ± 10.5 *	63.5 ± 7.7	0.01
Child-Pugh score	-	-	5.4 ± 0.5	8.1 ± 0.8 ^	11.5 ± 1.2 ^^§^	<0.0001
Weight (Kg)	71.5 ± 10.2	65.2 ± 8.2 *	66.4 ± 6.2 *	63.1 ± 6.1 *^	61.2 ± 6.7 *°^	<0.0001
Eight (cm)	163 ± 9	161 ± 10 *	162 ± 7	163 ± 7	161 ± 10	0.01
Body mass index (Kg/m^2^)	26.6 ± 2.2	25.1 ± 3.2 *	25.0 ± 2.7 *	23.7 ± 3.1 *°^	23.6 ± 3.2 *°^	<0.0001
Disease duration (yrs)	-	7.5 ± 4.2	9.6 ± 4.1 °	12.5 ± 4.0 °^	15.6 ± 4.1 °^^§^	<0.0001
Etiology						
Alcohol	-	1	7	6	6	-
Hepatitis B virus	-	21	11	10	10	-
Hepatitis C virus	-	90	44	42	45	-
Hepatitis B + D viruses	-	2	5	2	1	-
Hepatitis B + C viruses	-	14	-	2	4	-
Cryptogenic/metabolic	-	14	3	3	3	-
Plasma albumin (mg/dL)	4.51 ± 0.52	4.62 ± 0.61	4.01 ± 0.72 *°	3.32 ± 0.75 *°^	2.70 ± 0.62 *°^^§^	<0.0001
Serum creatinine (mg/dL)	0.71 ± 0.21	0.82 ± 0.32 *	0.82 ± 0.27	0.91 ± 0.30 *°	1.02 ± 0.32 *°^^§^	<0.0001
Total plasma bilirubin (mg/dL)	0.89 ± 0.12	0.95 ± 0.13	1.22 ± 0.62 *	2.52 ± 0.99 *°	3.51 ± 1.23 *°^	<0.0001
B-type natriuretic peptide (pg/mL)	25.7 ± 8.5	24.8 ± 11.7	33.5 ± 17.1	122.2 ± 31.5 *°^	137.3 ± 43.1 *°^	<0.0001
Mean blood pressure (mmHg)	98.2 ± 5.2	98.5 ± 6.5	97.1 ± 5.5	96.2 ± 6.5	91.1 ± 5.1 *°^^§^	<0.0001
Heart rate (beats/min)	57.2 ± 10.1	60.1 ± 7.2 *	63.3 ± 6.2 *°	67.2 ± 8.1 *°	73.3 ± 9.1 *°^^§^	<0.0001

Data as expressed as mean ± SD; *: *p* < 0.05 vs. controls; °: *p* < 0.05 vs. CH patients; ^: *p* < 0.05 vs. Child A patients; ^§^: *p* < 0.05 vs. Child B patients.

**Table 2:** Echocardiographic and haemodynamic measurements in control subjects, patients with chronic hepatitis (CH) and patients with cirrhosis categorised on the basis of Child–Pugh score.

	Control Group (no. 168)	CH Group (no. 142)	Child A Group (no. 70)	Child B Group (no. 65)	Child C Group (no. 69)	*p*
Left atrium A-*p* dimension (mm)	3.6 ± 0.5	3.6 ± 0.6	4.2 ± 0.6 *°	4.4 ± 0.5 *°	4.5 ± 0.4 *°^	<0.0001
Left atrium volume/BSA (mL/m^2^)	25.2 ± 5.2	26.5 ± 5.1	32.2 ± 2.2 *°	33.5 ± 5.6 *°	37.5 ± 5.5 *°^^§^	<0.0001
LV posterior wall thickness (mm)	8.8 ± 1.1	8.8 ± 1.0	9.1 ± 1.2	9.4 ± 1.2 *°	9.2 ± 1.0	0.0003
IV septum thickness (mm)	8.9 ± 1.1	8.8 ± 1.1	9.5 ± 1.2 *°	9.5 ± 1.1 *°	9.5 ± 1.2 *°	<0.0001
LV diastolic volume/BSA (mL/m^2^)	67.5 ± 7.7	68.2 ± 6.5	69.5 ± 7.2	70.5 ± 5.7 *	71.2 ± 6.7 *°	0.0006
LV systolic volume/BSA (mL/m^2^)	30.3 ± 6.3	31.2 ± 2.5	30.0 ± 7.6	31.1 ± 6.5	30.1 ± 5.7	0.42
LV mass (g/m^2.7^)	35.3 ± 3.3	41.3 ± 4.2 *	43.3 ± 3.9 *°	43.3 ± 3.5 *°	48.5 ± 4.5 *°^^§^	<0.0001
LV ejection fraction (%)	57.1 ± 5.5	56.2 ± 4.9	57.2 ± 5.2	55.1 ± 2.7	56.2 ± 5.6	0.05
Cardiac output (L/min)	3.2 ± 0.3	3.3 ± 0.2	3.5 ± 0.3 *°	4.0 ± 0.3 *°^	4.1 ± 0.2 *°^	<0.0001
e/a	1.11 ± 0.21	1.20 ± 0.31	0.91 ± 0.22 *°	0.81 ± 0.21 *°	0.71 ± 0.31 *°^	<0.0001
IVRT (ms)	86.1 ± 12.2	89.2 ± 12.0	101.3 ± 16.1 *°	114.1 ± 14.2 *°^	126.5 ± 15.1 *°^^§^	<0.0001
Deceleration time (ms)	198 ± 21	207 ± 16	235 ± 18 *°	257 ± 22 *°^	279 ± 25 *°^^§^	<0.0001
Aortic valve regurgitation (n; %)	19 (11.3)	16 (11.3)	9 (12.8)	8 (12.3)	9 (13.0)	0.94
Aortic valve stenosis (n; %)	5 (3.0)	5 (3.5)	4 (5.7) *°	4 (6.1) *°	5 (7.2) *°	0.03
Mitral valve regurgitation (n; %)	65 (38.7)	56 (39.4)	30 (42.8)	39 (60.0) *°^	42 (60.9) *°^	<0.0001
Mitral valve stenosis (n; %)	0 (0)	0 (0)	0 (0)	1 (0.01)	0 (0)	-
Mitral valve calcification (n; %)	131 (78.0)	114 (80.3)	62 (88.6)	60 (92.3) *°	65 (94.2)*°	0.04
Mitral valve calcification score	0.86 ± 0.25	0.85 ± 0.41	0.87 ± 0.30	1.65 ± 0.37 *°^	1.72 ± 0.35 *°^	<0.0001
ePSP (mmHg)	23.3 ± 6.3	22.5 ± 5.5	23.8 ± 6.1	28.8 ± 6.2 *°^	30.5 ± 5.1 *°^	0.02
SVR (dyn·s·cm^−5^)	2337 ± 344	2211 ± 368 *	1995 ± 311 *°	1701 ± 291 *°^	1550 ± 286 *°^	<0.0001

Data are expressed as mean ± SD; *: *p* < 0.05 vs. controls; °: *p* < 0.05 vs. CH patients; ^: *p* < 0.05 vs. Child A patients; ^§^: *p* < 0.05 vs. Child B patients. A-*p*: antero-posterior; BSA: body surface area; ePSP: estimated values of pulmonary artery systolic pressure; LV: left ventricle; IV: interventricular septum; IVRT: isovolumetric relaxation time; SVR: systemic vascular resistance.

**Table 3:** Main clinical, laboratory, haemodynamic and echocardiographic characteristics in cirrhotic patients with (+) and without (−) mitral valve regurgitation.

	Regurgitation + (n 111)	Regurgitation − (n 93)	*p*
Sex (M/F)	66/60	50/28	-
Age (yrs)	64.1 ± 11.2	64.5 ± 10.5	0.77
Child–Pugh score	10.2 ± 1.1	6.2 ± 0.7	<0.0001
Body mass index (Kg/m^2^)	23.6 ± 3.1	24.1 ± 2.8	0.23
Disease duration (yrs)	14.5 ± 4.0	10.7 ± 4.0	<0.0001
Plasma albumin (mg/dL)	2.91 ± 0.70	3.72 ± 0.61	<0.0001
Serum creatinine (mg/dL)	0.97 ± 0.31	0.86 ± 0.27	0.008
Total plasma bilirubin (mg/dL)	3.10 ± 1.12	1.35 ± 0.77	<0.0001
Mean blood pressure (mmHg)	93.1 ± 5.5	96.6 ± 5.7	<0.0001
Heart rate (beats/min)	71.2 ± 8.5	64.7 ± 7.0	<0.0001
Left atrium volume/BSA (mL/m^2^)	36.7 ± 5.5	32.7 ± 4.5	<0.0001
Left ventricular mass (g/m^2.7^)	46.7 ± 4.2	43.3 ± 3.7	<0.0001
Cardiac output (L/min)	4.1 ± 0.3	3.7 ± 0.3	<0.0001
e/a	0.77 ± 0.31	0.87 ± 0.21	0.008
IVRT (ms)	123.2 ± 14.7	107.1 ± 15.2	<0.0001
Deceleration time (ms)	272 ± 25	241 ± 17	<0.0001
Mitral valve calcification score	1.69 ± 0.36	1.15 ± 0.25	<0.0001
SVR (dyn·s·cm^−5^)	1601 ± 292	1895 ± 315	<0.0001

Data as expressed as mean ± SD BSA: body surface area; IVRT: isovolumetric relaxation time; SVR: systemic vascular resistance.

**Table 4:** Univariate and multivariate analyses of the main factors potentially associated with mitral valve regurgitation in cirrhotic patients.

	Univariate Analysis			Multivariate Analysis		
	OR	95% CI	*p*	OR	95% CI	*p*
Child-Pugh score	17.0	4.0–72.7	0.0001	19.9	5.1–115.7	0.0003
Disease duration	1.15	1.1–1.2	<0.0001	1.14	1.07–1.25	0.0004
Plasma albumin	0.20	0.05–0.94	0.04	0.87	0.11–64.04	0.95
Serum creatinine	1.55	0.88–2.73	0.15	-	-	-
Total bilirubin	1.50	1.22–1.85	0.02	0.65	0.35–1.32	0.22
Mean blood pressure	1.005	1.001–1.006	0.01	1.025	0.97–1.07	0.97
Heart rate	21.5	2.73–168.2	0.004	3.34	0.11–111.1	0.54
Left atrium volume *	3.12	1.87–5.17	<0.0001	3.2	1.5–6.5	0.0009
Left ventricle mass	1.07	1.02–1.12	0.0004	1.07	1.005–1.10	0.03
Cardiac output	2.12	1.17–3.81	<0.0001	0.9	0.5–3.4	0.85
e/a	0.82	0.71–0.96	0.008	0.89	0.65–1.25	0.78
Isovolumetric relaxation time	1.03	1.008–1.042	0.001	1.03	0.99–1.05	0.07
MV calcification score	6.15	2.49–15.43	<0.0001	12.5	2.39–68.07	0.005
Systemic vascular resistance	0.27	0.11–0.65	0.004	1.6	0.2–15.1	0.63

*: indexed to body surface area; MV: mitral valve.

## Data Availability

All data are available upon specific request to the first author.
